# The darkness of reacculturation: examining factors influencing depression among Chinese international student returnees in the post-COVID-19 era

**DOI:** 10.3389/fpsyg.2024.1407742

**Published:** 2024-06-19

**Authors:** Ruining Jin, Jie Wei, Xuanyi Wang, Tam-Tri Le

**Affiliations:** ^1^Civil, Commercial and Economic Law School, China University of Political Science and Law, Beijing, China; ^2^Suzhou Lunhua Education Group, Suzhou, China; ^3^Independent Researcher, Ho Chi Minh City, Vietnam

**Keywords:** returnees, Chinese international students, reacculturation, mental health, depression, cultural identity

## Abstract

**Introduction:**

In the aftermath of the COVID-19 pandemic, the reintegration of Chinese international student returnees intersects with China’s critical effort to combat a significant brain drain of highly skilled talents, highlighting an unprecedented psychological battleground. This convergence underscores the urgent need for nuanced research to navigate the uncharted territory of their mental health.

**Methods:**

Employing Bayesian analysis supported by Markov Chain Monte Carlo (MCMC) algorithms, this study examined depression prevalence and associated factors among 1,014 Chinese returnees. The sample comprised 44.87% male participants (455), 51.58% female participants (523), and 3.55% identifying as “Others” (36), with an age distribution of 61.74% aged 18-30 (626), 28.80% aged 31-40 (292), and 9.47% aged 41-50 (96). The mean age of participants was 29.6 years, ranging from 18 to 50 years. PHQ-9 diagnoses revealed alarming levels of depression, with 47.9% exhibiting a moderately severe degree of depressive disorder.

**Results:**

Our findings highlight the intricate interplay between identity clusters— “homestayers” (those leaning towards a Chinese cultural identity), “navigators” (those with a bicultural identity), and “wayfarers” (those leaning towards a foreign cultural identity)—and the likelihood of depression. Specifically, homestayers showed a clear and strong negative association, navigators demonstrated a positive association, and wayfarers had a positive yet unclear correlation with depression levels. Furthermore, acculturation, age, and gender showed no significant effects, whereas education mildly mitigated depression.

**Discussion:**

Based on these findings, we suggest the implementation of better-tailored mental health support and policies to facilitate smoother reintegration.

## 1 Introduction

### 1.1 Acculturation and reacculturation

Acculturation, coined by Redfield et al. (1936), is a process that occurs when groups with different cultures interact closely, leading to changes in their cultural patterns. Subsequently, the definition of acculturation has been expanded by follow-up studies as a dual process involving cultural and psychological transformation due to interactions among different cultural groups and their members, as it covers shifts in social structures, institutions, cultural practices, and a diverse range of individual behaviors (Berry, 1997, 2005; Sam and Berry, 2006). This ongoing process can last for a long time, from years to centuries, involving various forms of mutual adjustments leading to psychological and societal adaptations for both parties involved in the interaction (Sam and Berry, 2006).

Reacculturation, on the other hand, is a vital part of the reverse culture shock process when sojourns return to the environment they once lived. It involves the essential process of readjusting, reintegrating, and reassimilating into their home culture after living in a different cultural environment for a significant period (Gaw, 2000). Reacculturation varies from acculturation in several ways and can result in difficulties and disorientation (Adler, 1975; Adler, 1981). Initially, returnees may encounter unforeseen challenges due to mismatched expectations about reintegration. The place they once knew as their home may have changed during their absence, which could be a stressor for returnees. Additionally, individuals may experience identity changes while abroad, making it difficult to reintegrate into their home culture. Additionally, returning to their home country could result in a loss of status for individuals, leading to a decrease in the autonomy and respect they previously enjoyed abroad. This can contribute to the stress of readjusting to their original culture (Adler, 1975). Additionally, social reintegration and the challenges of reverse culture shock can lead to psychological disorientation and mental distress for returnees (Gaw, 2000).

### 1.2 Acculturative stress and its associated factors in reacculturation

Because of the hardships during acculturation and reacculturation, the term acculturative stress was coined to reflect many mental and psychological distresses during the process when returnees are confronted with the loss of social support, self-esteem, and identity, and perceived discrimination (Smart and Smart, 1995; Revollo et al., 2011). Prior studies on reacculturation have highlighted several factors associated with acculturative stress. At the micro level, individual personalities and cultural proficiency significantly determine the extent of acculturative stress experienced by returnees (Kidder, 1992; Presbitero, 2016). In East Asia or other hierarchical societies, returnees who struggle to conceal their emotions or conform to group norms tend to experience higher levels of acculturative stress (Kidder, 1992; Takeuchi et al., 2001; Jiang et al., 2015; Lee, 2016). Additionally, strained relationships with family members can exacerbate acculturative stress due to “communication breakdown” (Lee and Friedlander, 2014), and cultural value conflicts (Christou, 2003; Chang, 2010; Hwang S. S. et al., 2010). At the organizational level, returnees often experience acculturative stress when their high expectations for career advancement or work-life balance clash with the hierarchical organizational culture, hectic work schedules, and intense workloads they encounter (Pham and Saito, 2019; Liu et al., 2022; Rahimi et al., 2024). At the macro level, acculturative stress can arise from ideological conflicts between the returnees’ liberalized views and the more traditional and conservative culture of their home countries (Butcher, 2002; Schwartz et al., 2009; Gu and Schweisfurth, 2015; Shaheem, 2015; Ai and Wang, 2017).

### 1.3 Acculturative stress and mental health among Chinese returnees in the post-COVID-19 era

While it is intuitive to assume that the above individual-subjective and environmental-factors-culminated acculturative stress would be negatively associated with migrants’ mental health conditions, prior studies suggested a complexity of interplay. Earlier studies suggested that acculturative stress is associated with a wide array of psychological problems, such as anxiety, depression, and identity confusion (Berry et al., 1987). However, recent studies such as Khan (2015)‘s literature review based on 20 relevant studies indicated that contradictory findings were found between acculturative stress and mental health; some even challenged the conventional wisdom, concluding that during COVID-19, more frequent racist encounters were linked with a perceived improvement of participants’ health conditions (Nie, 2024); Liu et al. (2016)‘s study on Chinese international students is consistent with earlier traditional conclusion that acculturative stress components were correlated to depression, while Li and Goldman (2024)‘s conclusion offered a more balanced view on this issue, addressing the importance of the level of acculturation on acculturative stress reflected by friendship instability.

For Chinese international students, many faced acculturation challenges such as language proficiency and social alienation (Choy and Alon, 2019), academic and financial hardship (Liu, 2009), cultural adjustment and face concern (Magnusdottir and Thornicroft, 2022). A prior study has indicated that immigrants’ subjective well-being is positively associated with their acculturation level in the West (Güler and Yildirim, 2021), so the above mentioned acculturation challenges might lead to decreased subjective well-being, reflected by mental health issues such as depression and anxiety symptoms (Lian and Wallace, 2018). During the COVID-19 pandemic, research has shown that Chinese international students experienced a high prevalence of mental health issues, specifically depression and anxiety (Anan et al., 2020; Lai et al., 2020; Li et al., 2021; Hu et al., 2023; Chen et al., 2024). However, to those who chose to return China during the initial outbreak of COVID-19, their mental health issues persisted. First of all, they went through a rational but involuntary displacement from their host country to China (Zhang et al., 2022). Earlier studies have concluded that involuntary migration would lead to mental distress among migrants (Hwang et al., 2007; Anbesaw et al., 2024). Secondly, their reentry was coupled with escalated Sino-US tension and Cybernationalism in China, where individuals with transnational and cosmopolitan identities were forced to take a stand between the China “us” and the West “them” (Catalano and Wang, 2021; Yu, 2021). In this context, those with an individualistic value prioritization or those who defied quarantine prevention measures were stigmatized and discriminated against (Jin and Wang, 2022). A prior study on forcibly displaced immigrants suggested that perceived discrimination significantly diminishes personal wellbeing of this population (Güler and Yıldırım, 2022).

As the global COVID-19 pandemic subsided, Chinese international students returning to China faced the complexities of reacculturation and new societal and economic pressures. During the pandemic, measures such as lockdowns and “Normalized Prevention Control” implemented by the Chinese government further slowed economic momentum (Liu, 2023). The Chinese government officially ended its “Zero-COVID” policy in December 2022. Consequently, early 2023 saw high expectations for a potent economic bounce-back in the post-COVID-19 era (SCMP, 2023). However, the economic recovery fell short of expectation (Ajami and Karimi, 2023). Numerous socioeconomic crises that emerged during COVID-19 persisted, including a slow economic growth (Attinasi et al., 2023; IMF, 2023; Luo and Li, 2023; Song, 2023), rising youth unemployment (ICEF, 2023), high divorce rate (SCMP, 2024), and a significant exodus of skilled labor (Bloomberg News, 2023; Li, 2023; Wong and Yan, 2023).

These socioeconomic crises might impose an additional burden on returnees’ mental health as they start their careers. Additionally, from a sociocultural perspective, Chinese society has taken a “right” turn toward conservatism, marked by a more assertive diplomatic stance, increased centralized government control, and efforts to revive traditional values to bolster national confidence and achieve the “great rejuvenation of the Chinese nation” (Iida, 2020; Blanchette and Medeiros, 2022; Zhao, 2023). These social changes may conflict with Western progressive political agendas and liberal views, potentially causing identity conflicts and acculturative stress among returnees. Moreover, due to the abovementioned political shift and slow economic growth, labor rights infringements have become pervasive in the Chinese workplace, with average weekly working hours reaching 48 (CEIC, 2024). It is evident that returnees who previously lived or studied in developed countries might experience identity conflicts and acculturative stress due to the hectic work schedules and hierarchical workplace culture (Pham and Saito, 2019; Liu et al., 2022).

Prior studies on Chinese returnees’ reacculturation, although not explicitly focusing on mental health, might offer some insights into their acculturative stress. Several studies shed light on factors that caused returnees’ acculturative distress in the post-COVID era in China, such as work-life balance (Liu et al., 2022), cybernationalism and outgroup bias (Jin and Wang, 2022), and lack of autonomy in career development (Ma et al., 2023). However, limited research has focused on transnational identity clusters and how different sojourners’ transnational identity clusters might impact their acculturative distress during acculturation. This lack of focus is significant because these identity clusters can lead to varying levels of mental health concerns.

### 1.4 Chinese international student returnees’ various identity clusters

The formation of transnational identity is a common phenomenon among sojourners and migrants during their acculturation process. Interactions of values from foreign and domestic social environments may lead to groups of specific cultural identities being formed within the sojourner-migrant population. On the other hand, self-categorization is the psychological mechanism of how a person perceives as belonging to a collective group of certain social values and expressions (Turner et al., 1987). When a social identity is established, corresponding perceptions and expectations are formed and reinforced in one’s mind, which might be distinct and conflict with other groups’ values (Tajfel and Turner, 1979).

In the case of Chinese international student returnees who grew up in China and finished their overseas study, Wang (2022) terms “homestayers,” “wayfarers,” and “navigators” to symbolize how Chinese international student returnees navigate their identity between China and their host country. Homestayers are individuals who focus on integrating into their local Chinese environment and maintaining a predominantly Chinese-oriented identity; Wayfarers are those who intentionally distance themselves from their Chinese heritage and immerse themselves in exploring and embracing foreign cultures; Navigators are individuals who adeptly navigate the intricacies of both their native Chinese setting and foreign cultural impacts, leading to the formation of a mixed identity.

### 1.5 Current study

In light of the profound social and economic disturbances characterizing the post-COVID-19 landscape in China, it is critical to explore the mental health implications for Chinese returnees. This demographic is uniquely positioned at the intersection of reacculturation challenges and the broader pandemic-induced crises, facing not only the standard hurdles of adjusting back to their home culture but also the added strain of an altered societal and economic environment. Delving into these mental health concerns is essential for pinpointing specific vulnerabilities, tailoring support mechanisms, and developing robust policies aimed at facilitating smoother reintegration. Furthermore, this inquiry promises to enrich the global discourse on managing the psychological repercussions of large-scale crises, offering insights into resilience-building and mental well-being maintenance in tumultuous times. To these ends, the current study has two research objectives:

1. Examining the prevalence of depression within the Chinese returnee population.
2. Identifying factors contributing to depression among this population.

## 2 Methodology

### 2.1 Materials and variables

A total of 1,014 Chinese international student returnees participated in the survey, which was conducted through WeChat Chinese international student returnee public groups in the following cities: Beijing, Shanghai, Suzhou, Shenzhen, and Guangzhou. The researchers gained access to these groups by searching international student returnee-related keywords in the WeChat Public Account search. After subscribing to the relevant public accounts, the researchers’ returnee status was verified, allowing them to enter the groups to distribute informed consent forms, explain the purpose of the study, and share the link to the online survey. Due to the sensitive nature of the study, the names of the public groups and accounts will not be disclosed to ensure the safety and security of all participants. Group sizes varied between 200 and 500 users, with two groups composed of US-based returnees, two of UK-based returnees, and one of Australia-based returnees. Data collection for the survey occurred from October 8, 2023, to January 30, 2024. Considering the notion that individuals undergoing culture shock and reverse culture shock typically undergo a “honeymoon” phase (Oberg, 1954), which may momentarily alleviate the intensity of acculturative stress; and the possibility that respondents will join various city-based WeChat public groups for returnees, the following criteria constitute the inclusion criteria for this survey: (1) born and raised in China and went overseas for an educational purpose; (2) returned to China after studying abroad; (3) resided in China for at least 1 year after returning; and (4) have not taken part in the same survey in other WeChat public groups. It is important to consider that there have been significant movements of highly skilled Chinese laborers leaving China for Western countries in recent years, so this data collection also included those who were not physically present in China when they completed the survey, as long as they met the abovementioned criteria. The survey questions were reflected in the WeChat MiniApp SurveyStar. Subsequently, the researchers provided the study’s objectives, informed consent, survey link, and recruitment criteria to the WeChat returnee public groups. The final valid sample consisted of 1,014 participants after multiple rounds of screenings, including the removal of single responses for all questions and short-time answers (within 60 s). Out of the 1,014 participants, 455 responses were from male participants, representing roughly 44.87% of the total. There are 523 female respondents, accounting for about 51.58%. Additionally, 36 respondents (about 3.55%) identified as “Others” in this category. Out of 1,014 participants, 61.74% were aged 18–30 (626 participants), 28.80% were in the 31–40 age group (292 participants), and 9.47% were in the 41–50 age group (96 participants). The data collection was authorized by the Institutional Review Board of the first author’s institution. The survey was conducted anonymously and did not include any details that could reveal the participants’ identities. Informed consent was obtained from all participants before they took part in the study. Table 1 displays the variables utilized in this analysis from the dataset.

**Table 1:** Variable description.

Variable name	Meaning	Variable type	Value
Depression	The participant’s total PHQ-9 score	Numeric	Ranging from 0 to 27
Homestayer	Whether the participant is self-identified as a homestayer	Binary	0. No 1. Yes
Navigator	Whether the participant is self-identified as a navigator	Binary	0. No 1. Yes
Wayfarer	Whether the participant is self-identified as a wayfarer	Binary	0. No 1. Yes
Adapt	The participant’s average SAS score	Numeric	Ranging from 1 to 5
Edu	The participant’s highest educational attainment	Numeric	1. Elementary or lower 2. Secondary School 3. High School 4. Undergraduate 5. Postgraduate or higher
Age	The participant’s age group	Numeric	1. <18 2. 18–30 3. 31–40 4. 41–50 5. >50
Male	The participant self-identifying as a male	Binary	0. No 1. Yes
Female	The participant self-identifying as a female	Binary	0. No 1. Yes
Other	The participant self-identifying as a different gender from either male or female	Binary	0. No 1. Yes

*All participants in this sample are over 18 years old.

The variable *Depression* is a numeric variable with its value being the participant’s total score (ranging from 0 to 27) on the Patient Health Questionnaire-9 (PHQ-9) diagnosis (Kroenke et al., 2001). The binary variables *Homestayer, Navigator,* and *Wayfarer* were created based on participants’ self-identification into one of the three identity clusters. Definitions and concise explanations of these three identity clusters (Wang, 2022) were presented next to the question. However, while Wang (2022)‘s qualitative study explored identity clusters beyond cultural belonging and preference by including dimensions like translocality, cross-cultural competence, and social capital, for the current quantitative study, the survey questions focused mainly on cultural preference and belonging between home (China) and host (foreign) country to ensure participants’ understanding. Participants were directed to select only one answer from the three identity clusters. The variable *Adapt* represents the participant’s average Short Acculturation Scale (SAS) score (Marin et al., 1987; Gupta and Yick, 2001) ranging from 1–5, where a higher value means a higher degree of acculturation. The questions of SAS were adapted to the context of this study. The variable *Edu* refers to the participant’s highest educational attainment. The variable *Age* reflects the participant’s age group. Note that all participants in this sample are over 18 years old. Lastly, the binary variables *Male, Female,* and *Other* were created based on participants’ self-identification into one of the three gender identities. Participants were directed to select only one option from the three gender identities.

### 2.2 Analysis procedure

In the current study, Bayesian analysis was employed, aided by Markov Chain Monte Carlo (MCMC) algorithms. Model construction, analysis procedure, and result presentation follow the protocols of MCMC-aided Bayesian analytics for social sciences (Nguyen et al., 2022; Vuong et al., 2022). The formula of the analytical model is as follows.


$$\begin{array}{l} \mu_{i}=\beta_{0}+\beta_{\mathrm{Homestayer}}*\mathrm{Homestaye}r_{i}+\beta_{\mathrm{Navigator}}*\mathrm{Navigato}r_{i}+\\ \beta_{\mathrm{Wayfarer}}*\mathrm{Wayfare}r_{i}+\beta_{\mathrm{Age}}*Age_{i}+\beta_{Edu}*Edu_{i}+\\ \beta_{\mathrm{Adapt}}*\mathrm{Adap}t_{i}+\beta_{\mathrm{Male}}*Male_{i}+\\ \beta_{\mathrm{Female}}*\mathrm{Femal}e_{i}+\beta_{\mathrm{Other}}*\mathrm{Othe}r_{i} \end{array}$$

$\mu_{i}$ is the mean value of participant i ’s degree of depression (outcome variable *Depression*) with posterior estimations in the form of normal distribution. Participant i ’s perceived China-leaning identity cluster status is measured by $\mathrm{Homestaye}r_{i}$ . Participant i ’s perceived bicultural identity status is reflected as $\mathrm{Navigato}r_{i}$ , and participant i ’s status of perceived West-leaning identity is $\mathrm{Wayfare}r_{i}$ . Participant i ’s age group is $Age_{i}$ . Participant i ’s educational attainment is $Edu_{i}$ . Participant i ’s acculturation degree is measured by $\mathrm{Adap}t_{i}$ . Participant i ’s perceived gender identity of being male is measured by $Male_{i}$ . Participant i ’s perceived female identity is $\mathrm{Femal}e_{i}$ . Participant i ’s self-identified gender identity that is different from either male or female is $\mathrm{Othe}r_{i}$ . The model has an intercept $\beta_{0}$ and coefficients $\beta_{\mathrm{Homestayer}}$ , $\beta_{\mathrm{Navigator}}$ , $\beta_{\mathrm{Wayfarer}}$ , $\beta_{\mathrm{Age}}$ , $\beta_{Edu}$ , $\beta_{\mathrm{Adapt}}$ , $\beta_{\mathrm{Male}}$ , $\beta_{\mathrm{Female}}$ , $\beta_{\mathrm{Other}}$ .

MCMC-aided Bayesian analysis has statistical advantages when working with a relatively small sample size, MCMC processes can help minimize inherent skewness of values within the sample, as well as improve inference accuracy given the effective sample size is healthy. Chinese international student returnees are a special population, and it is not easy to gather very large samples of them. Additionally, when examining derived variables such as the binary variables of gender in this study, the number of participants self-identifying as having a different gender than either male or female is quite small. Besides gender variables, identity cluster binary variables also present a low-data-point situation. MCMC algorithms can help generate a large number of simulated data points based on the original data and increase the estimation accuracy of the model’s posterior results.

The Bayesian approach considers all characteristics in a probabilistic manner. Results interpretation depends on parameters with the highest probability of occurrence in their posterior distributions. This can improve the accuracy of evaluation in psychological studies (Dunson, 2001; Csilléry et al., 2010; Gill, 2014; Wagenmakers et al., 2018). Markov chain convergence is evaluated by analyzing the effective sample size (*n_eff*) and the Gelman-Rubin shrink factor (*Rhat*). It is essential for the *n_eff* values to surpass 1,000 in this situation (McElreath, 2020). The *Rhat* values should ideally be 1 (Gelman and Rubin, 1992; Brooks and Gelman, 1998). The analysis was conducted using the bayesvl package in R (La and Vuong, 2019). Markov chain convergence can also be visually assessed by trace plots, Gelman-Rubin-Brooks plots, and autocorrelation plots. The influencing factors of depression of Chinese international student returnees in the post-COID-19 social context have not been well-studied before, thus non-informative priors were utilized in the analytical model to reduce initial subjective biases. The MCMC configuration consists of 5,000 total iterations, with 2000 warm-up iterations and 4 chains.

## 3 Result

**Figure 1:**
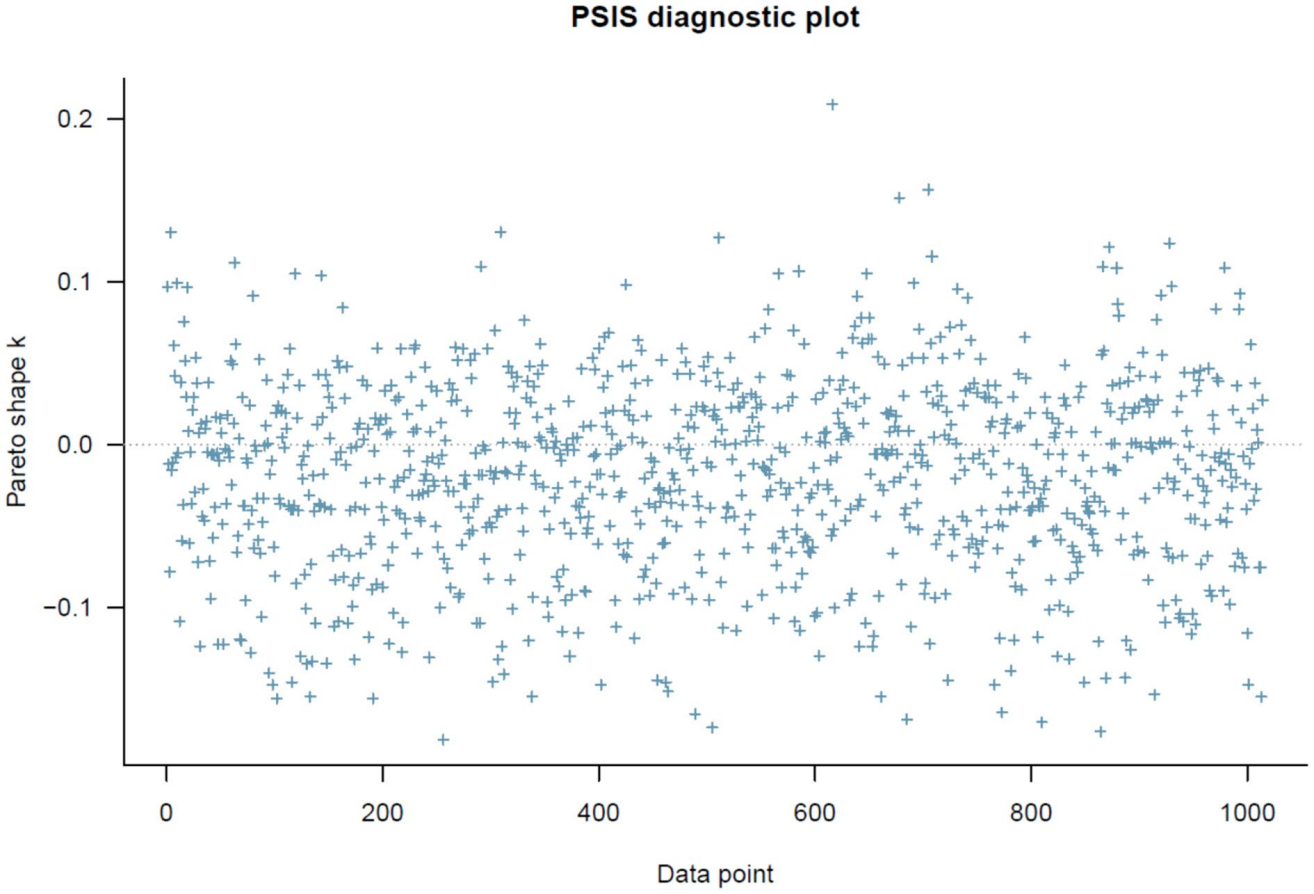
PSIS diagnostic plot.

Regarding the PHQ-9 diagnosis, depression severity is categorized into groups based on the total score (minimum is 0 and maximum is 27), namely: no depression (0 to 4), mild depression (5 to 9), moderate depression (10 to 14), moderately severe depression (15 to 19), and severe depression (20 to 27). In the current study’s sample (*N* = 1,014), the percentage of each depression severity group is as follows: 18.2% none (*N* = 185), 8.2% mild (*N* = 83), 20.1% moderate (*N* = 204), 47.9% moderately severe (*N* = 486), and 5.5% severe (*N* = 56) (Figure 1).

**Figure 2:**
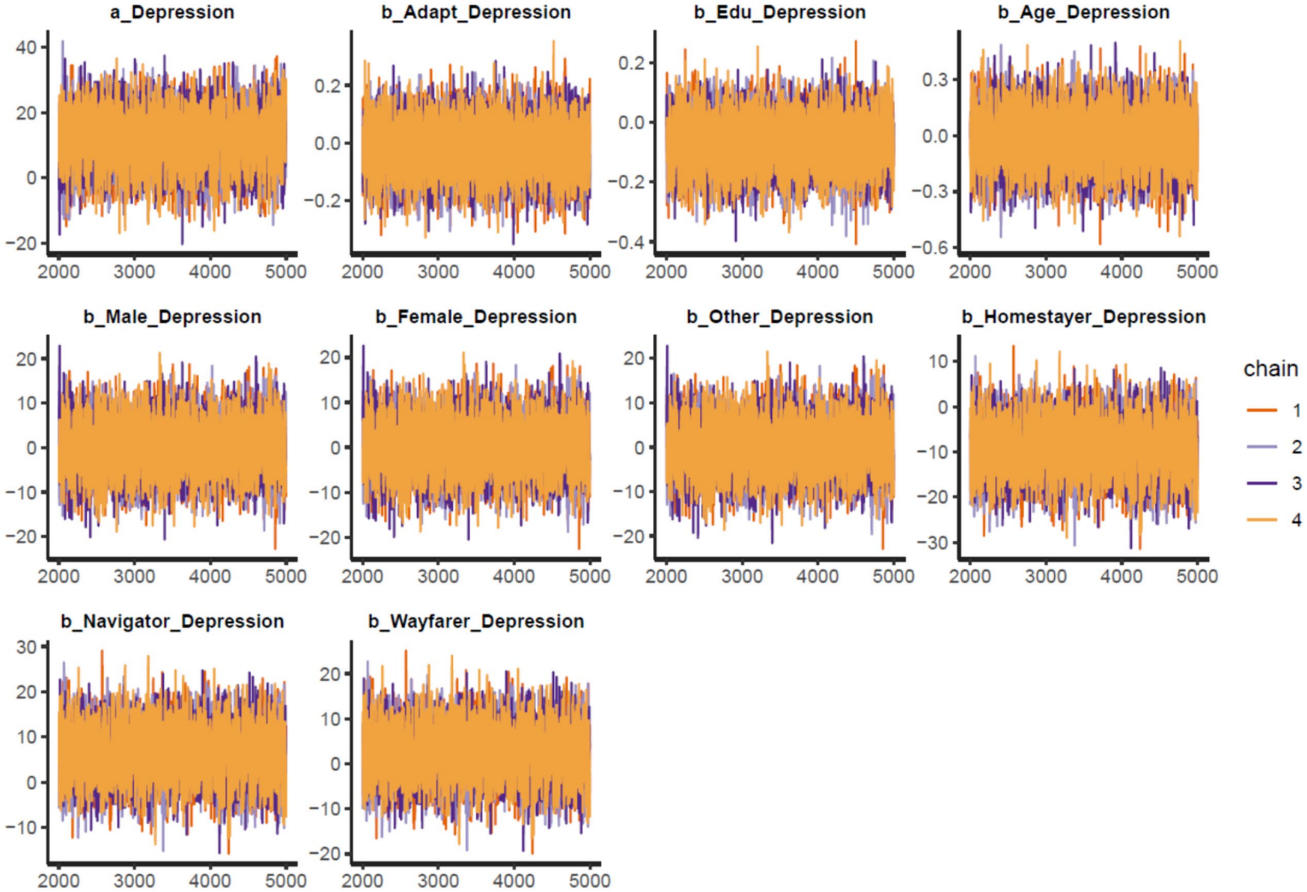
Trace plots.

Figure 2 below shows the PSIS diagnosis result. All *k* values are smaller than the threshold of 0.5, which could be seen as a goodness-of-fit sign.

The findings are presented in Table 2. The statistical analysis indicates that the model’s Markov chains have good convergence, since the effective sample size (*n_eff*) is above 1,000 and the Gelman-Rubin shrink factor (*Rhat*) is 1, demonstrating acceptable reliability of the posterior coefficients. Figure 2 displays trace plots showing colored lines representing the Markov chains. We can observe that lines fluctuated around a central equilibrium after the warmup period, and it is therefore a good indicator of well-mixing and stationary qualities.

**Table 2:** Simulated posteriors.

Parameters	Mean (M)	Standard deviation (S)	*n_eff*	*Rhat*
Constant	11.28	8.22	4,560	1
Homestayer	−9.11	5.80	4,568	1
Navigator	6.57	5.80	4,576	1
Wayfarer	2.54	5.80	4,581	1
Male	0.18	5.79	5,523	1
Female	0.14	5.79	5,522	1
Other	0.02	5.80	5,535	1
Edu	−0.07	0.09	11,785	1
Age	−0.02	0.14	10,830	1
Adapt	−0.02	0.09	11,310	1

**Figure 3:**
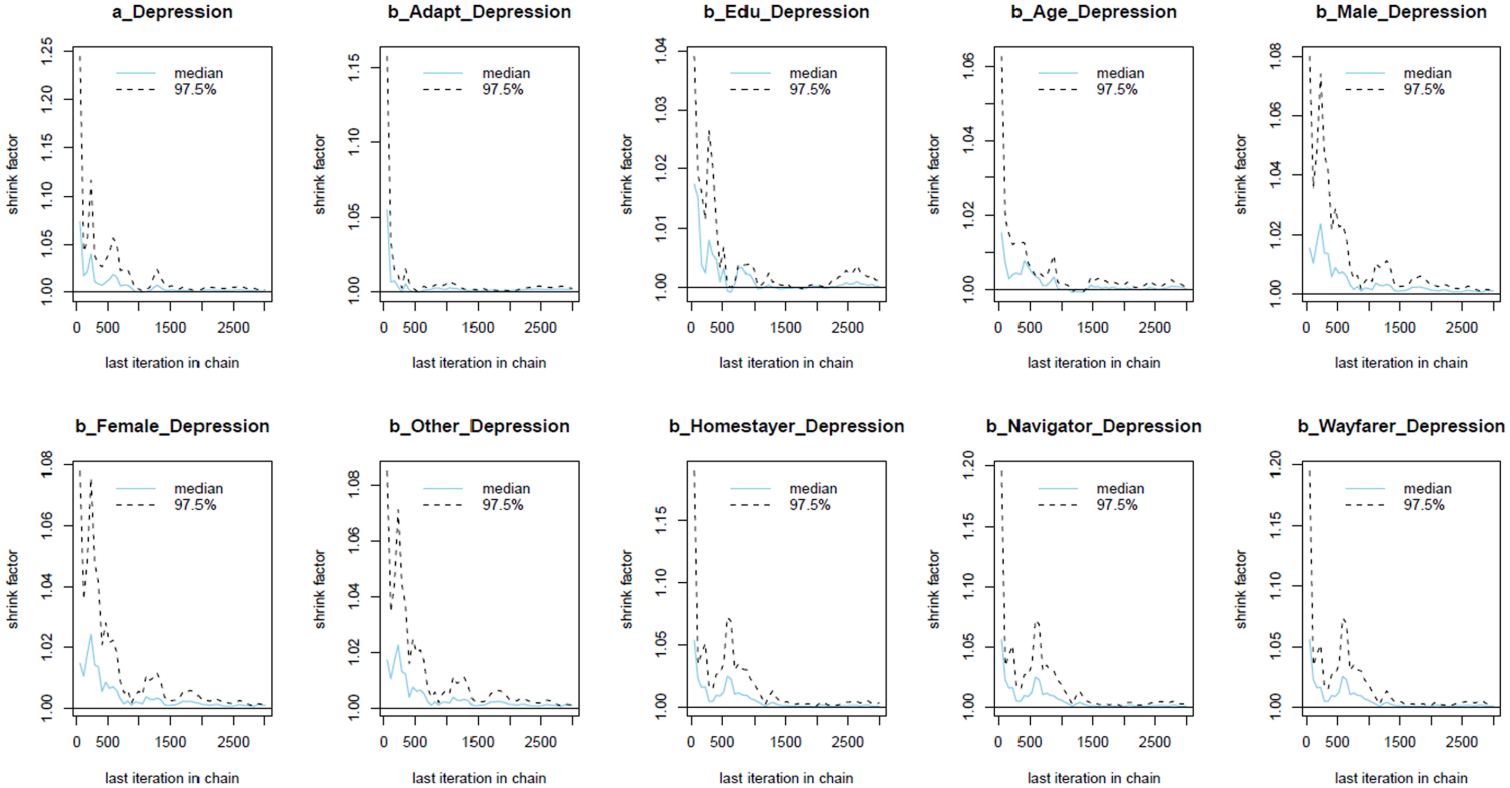
Gelman-Rubin-Brooks plots.

**Figure 4:**
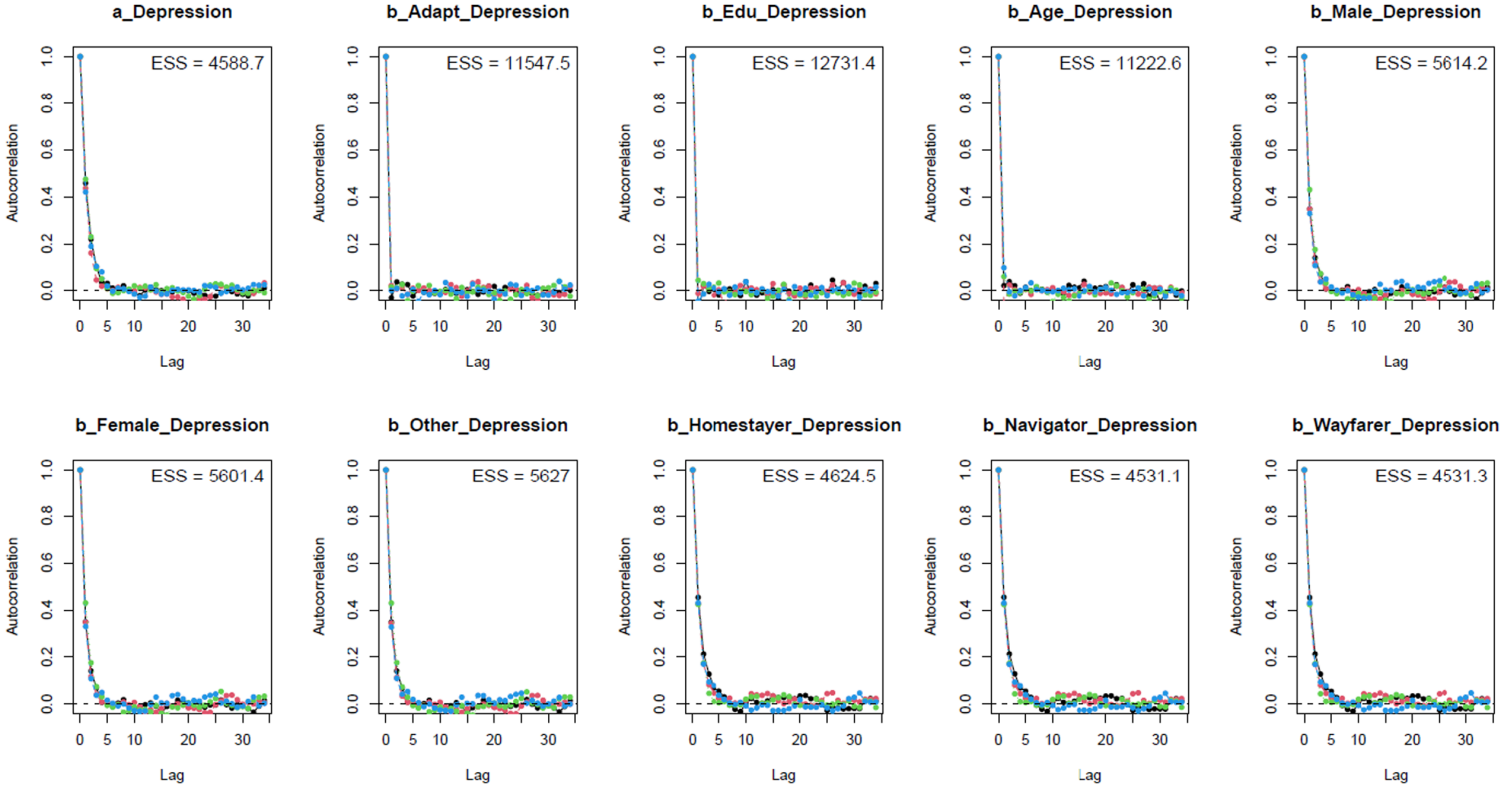
Autocorrelation plots.

The Gelman-Rubin-Brooks plots (Figure 3) illustrate that *Rhat* values decline quickly to 1 in the warm-up period. The autocorrelation plots (Figure 4) also suggest a quick elimination of problematic autocorrelation among simulated data points within the MCMC processes.

**Figure 5:**
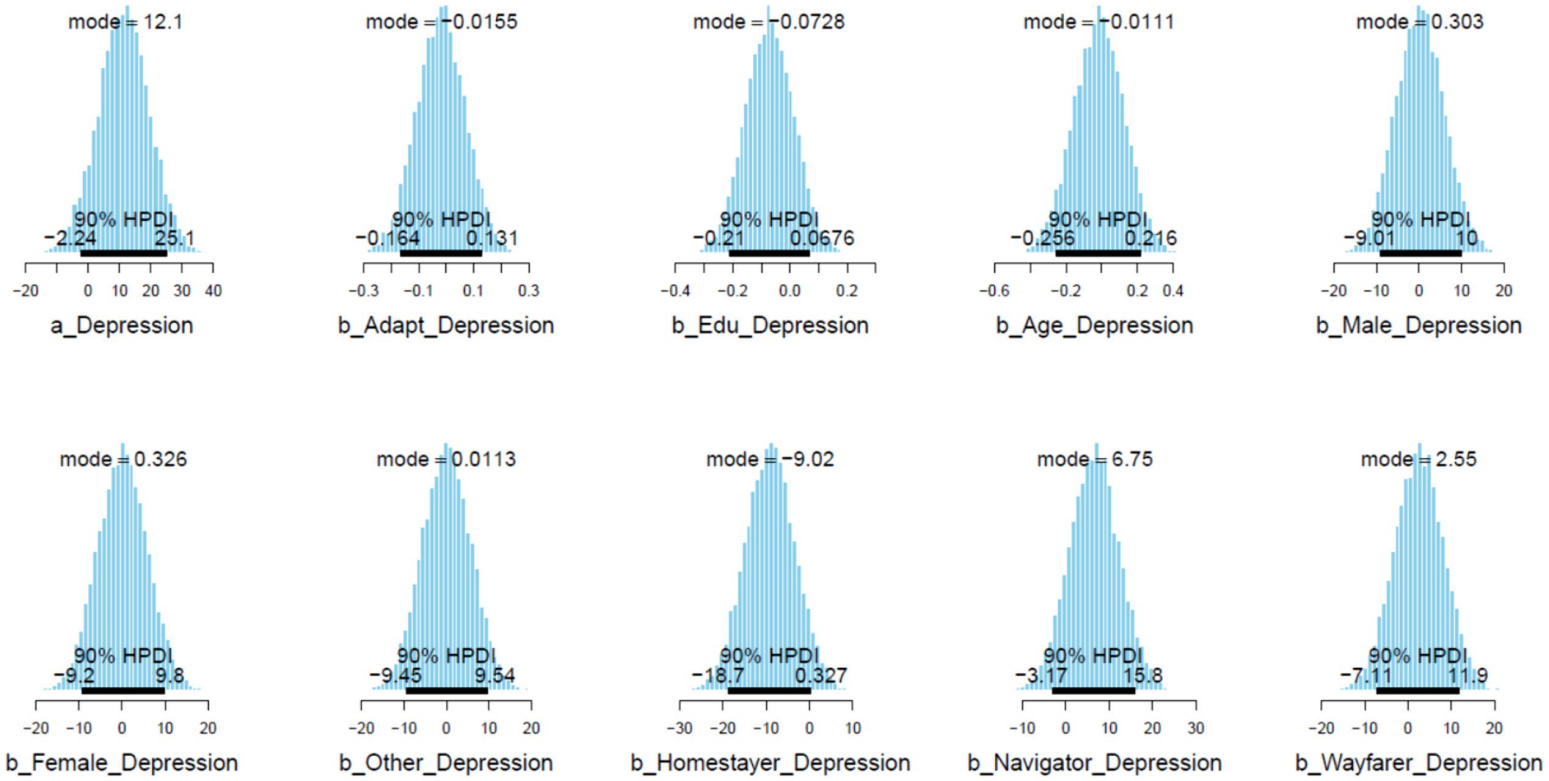
Posterior distributions within 90% of highest posterior density intervals (HPDIs).

According to the analysis results, identity cluster *Homestayer* has a clear negative association with depression ( $M_{\mathrm{Homestayer}}$ = −9.11 and $S_{\mathrm{Homestayer}}$ = 5.80), and identity cluster *Navigator* indicates a clear positive association with depression ( $M_{\mathrm{Navigator}}$ = 6.57 and $S_{\mathrm{Navigator}}$ = 5.80). Identity *Wayfarer* has a positive yet unclear association with depression ( $M_{\mathrm{Wayfarer}}$ = 2.54 and $S_{\mathrm{Wayfarer}}$ = 5.80). In addition, sociodemographic and sociocultural variables except education, such as *Age, Adapt, Male, Female,* and *Other* do not show any clear association with depression. ( $M_{\mathrm{Age}}$ = −0.02 and $S_{\mathrm{Age}}$ = 0.14, $M_{\mathrm{Adapt}}$ = −0.02 and $S_{\mathrm{Adapt}}$ = 0.09. $M_{\mathrm{Male}}$ = 0.18 and $S_{\mathrm{Male}}$ = 5.79. $M_{\mathrm{Female}}$ = 0.14 and $S_{\mathrm{Female}}$ = 5.79, $M_{\mathrm{Other}}$ = 0.02 and $S_{\mathrm{Other}}$ = 5.80). Educational attainment was found to be negatively associated with depression ( $M_{Edu}$ = −0.07 and $S_{Edu}$ = 0.09); however, the magnitude of the effect is quite low. In Figure 5, it can be observed that the posterior distributions of *Navigator* lie mostly on the positive side, while *Homestayer* is almost completely on the negative side. Moreover, the posterior distributions of *Edu* also mainly lie on the negative side.

## 4 Discussion

### 4.1 High prevalence of depression in Chinese returnees

The analysis results of PHQ-9 diagnosis results show an alarmingly high prevalence of depression in this Chinese returnee sample. Particularly, those with moderately severe depression (total score ranging from 15 to 19) accounted for nearly half of the sample size (47.9%). Indeed, the rising depression rate in China has been noticed in prior studies (Qin et al., 2016; Ren et al., 2020). However, compared with a prior study conducted by Qin et al. (2016) on the Chinese general population’s depression rate (37.9% for depressive symptoms and 4.1% for depression), and global lifetime depression prevalence of 10–15% (Lépine and Briley, 2011), the number is still striking. On the other hand, depression among returnees is higher than their domestic college student counterpart, as one meta-analysis study indicated that the overall ratio of depression in Chinese university students is 23.8% (Lei et al., 2016).

One possible explanation for the high prevalence of depression among Chinese returnees is the impact of COVID-19. The catastrophic impacts of COVID-19 pandemic on international students mental health have been well-documented in Iftikhar et al. (2022)‘s study, where it was found that international students in South Korea during the COVID-19 pandemic displayed sleep problems, anxiety, and depression rates at 47.1, 39.6, and 49%, respectively. Although a longitudinal study on depression, anxiety, and stress levels among young adults in India during COVID-19 suggested that these levels decreased when the lockdown ended (Rehman et al., 2023), our current study indicates otherwise. While China has officially abandoned its harsh “Zero-COVID” policy and thereafter eliminated all COVID-19-prevention measures such as hotel quarantine and daily COVID-19 testing by the end of 2022, the returnees might still suffer from the long-lasting influence of what happened during COVID-19. Namely, some of them might still live under stigmatization due to the notorious “double stigmatization,” during which returnees were stigmatized twice in the host country as well as China as a result of identity politics, political othering, and escalated China-West relationship (Yu, 2021; Jin and Wang, 2022). Such traumatic mental distress experience might culminate in depression for all Chinese international student returnees regardless of identity clusters. In addition, COVID-19 also hammered China’s economic momentum in various ways such as decoupling between China and the West, and the consequent manufacturing desolation (Witt et al., 2021; Attinasi et al., 2023) and fading domestic consumer confidence (Chen and Dong, 2022), which in turn, might bring about uncertainties toward future career and identity conflicts in a gradual nationalism and conservative-leaning society (Ferdinand, 2016; Sullivan and Wang, 2022). Also, COVID-19-induced slow economic growth may further deteriorate the exploitative workplace culture in developing countries, leading to mental distress among returnees who aim to seek work-life balance (Pham and Saito, 2019; Liu et al., 2022).

### 4.2 Depression and the demographic factors

The multi-regression analysis results show the relationships between depression and some demographic and psychological factors. We found that the degree of acculturation to the foreign country/countries does not have any clear association with returnees’ depression. Age also shows no significant correlation, which is consistent with an earlier study based on a cross-cultural setting (Brabant et al., 1990). Educational attainment was found to be negatively associated with depression (moderate reliability), suggesting a mitigating effect of education; however, the magnitude of this effect is small. No gender identifications (male, female, or other) were found to have any clear correlation with returnees’ depression. Regarding identity clusters, homestayers were found to have a clear and strong negative association with depression. On the other hand, navigators have a clear and strong positive association with depression. Wayfarers may have a slight positive association with depression, but this effect is unclear (low reliability).

While some past studies suggest that acculturative stress may lead to depression (Schwartz et al., 2010; Baker et al., 2012; Yoon et al., 2013), none has examined how the degree of acculturation in the host country may be associated with the mental health of returnee upon acculturation into the home country. The current study findings suggest that a high degree of acculturation (adaptation) to foreign environments likely does not affect the mental well-being of returnees, as human minds are flexible and can deal with the mental burden of reverse acculturation regardless of past experience of acculturation to foreign environments. One possible speculation is that most participants in this sample are relatively young (within labor age) and thus may be more mentally flexible compared to the cases of old returnees. However, we also found that age groups were not associated with depression. This implies a non-compromised adaptation, where being well-adapted to a new environment does not seem to create psychological conflicts toward re-adapting to the old environment. Considering the above, it is possible that acculturation can be considered to have the characteristics of a flexible new skill acquisition rather than being a rigid mindset-shifting process.

Speaking on the mitigating effect of education attainment against depression, it is intuitive to assume that more knowledge leads to greater skills and responding capabilities in the face of difficulties. This might help individuals gain culturally responsive and appropriate social support, which has been proven to be effective against acculturative stress and depression (Crockett et al., 2007). The findings that no differences were found among different gender identities suggest that gender is likely not a significant factor when considering depression in Chinese returnees. After all, male and female returnees both have a lot of social expectations upon coming back to China, including pressure from sexist stereotypes toward both genders (Qing, 2020; Liu et al., 2023; Ma et al., 2024; Rahimi et al., 2024); In addition, LGBTQ and other minority groups also face a lot of mental pressure in modern Chinese society (Wang and Bao, 2023). In brief, while returnees of each gender identification (male/female/other) face different challenges, there is not enough distinction that can influence depression likelihood in any clear pattern.

### 4.3 Depression and identity clusters

For homestayers, their identity cluster and the negative association with depression are very intuitive. This finding aligns with a prior study on sojourners in Nepal, which found that strong co-national identification can lead to better psychological well-being (Ward and Kennedy, 1999). As homestayers are influenced by a deep-rooted affinity for Chinese cultural and societal norms (Wang, 2022), they could encounter an intensification of cognitive dissonance and psychological discomfort stemming from encounters with foreign cultures that starkly contrast with their ingrained pro-China mindset. And when they returned to China, such internal conflicts could be mitigated. Moreover, the evolution of the sociopolitical landscape in China during their time abroad was marked by a pivot toward nationalism, centralization, and the reinforcement of central government authority (Leutert, 2018). Such a pivot might further amply the association upon their reentry as it resonates more profoundly with homestayers due to their heightened cultural and ideological congruence with a more “Chinese” society. As a result, homestayers are more likely to be considered part of the ingroup by locals, receiving a greater level of social support to cope with acculturative stress compared with the other two identity clusters. A prior study on Soviet Jewish integration into U.S. society also suggested that acculturation to the predominant culture is associated with a greater level of perceived social support from local peers (Birman et al., 2002).

Regarding navigators, their cross-cultural proficiency does not manifest their invulnerability to depression; instead, their identity cluster is positively associated with higher depression levels. Such a finding is inconsistent with prior studies regarding acculturation and mental distress (Jin and Wang, 2022; Wang, 2022; Li and Goldman, 2024). One possible explanation is that although self-identified as bicultural, they might actually run into more psychological conflicts compared with the other identity clusters. After all, they have to learn to draw the bottom line and balance conflicting worldviews and values. For example, in Western and more progressive ideologies and cultures, egalitarianism treats individuals in society as equal members (Rio et al., 2022), therefore by and large, the small power distance in the West between leaders and followers might become the sociocultural norm for navigators. However, in Eastern hierarchical societies such as China, the power distance is large between leaders and followers due to the concept that “the ruler being the principle of the followers” embedded in the Confucian “Three Principles” (Hofstede, 1984; King and Bond, 1985; Tan, 2015). Such a fundamental discrepancy in social structure and value prioritization between the West and China might cause identity conflicts and cognitive dissonances among navigators (Liu et al., 2022; Ma et al., 2023), which has been well-documented by other studies on returnee’s mental health challenges when returning to traditional hierarchical societies after experiencing more egalitarian cultures abroad (Brabant et al., 1990).

The popular “996” work schedule (referring to 9 A.M. – 9 P.M., 6 days a week) is a reflection of such a hierarchical workplace culture and employer-employee power imbalance. When navigators went through the acculturation process and realized that such a policy has been adopted by many Chinese companies and eventually became the industrial norm for many (Fan, 2023), they would face the dilemma of taking a side between sticking to individual freedom/better life quality and adhering to the collective expectations/questionable workplace regulations. Either choice might partially confront their cultural identity, leading to cognitive dissonance and identity conflicts among this population. Such a speculation has been corroborated by prior studies on returnees’ felt acculturative stress and the perceived exploitative workplace culture (Pham and Saito, 2019; Liu et al., 2022). Thus, while said to be the “flexible” cluster, in reality, they might face a lot of internal burdens from the expectation of balancing and dealing with such conflicting values, and therefore they are prone to a higher likelihood of depression.

With regard to wayfarers, the study findings suggest an unclear association in the direction of higher depression likelihood (low reliability). One possible explanation for this result is that their return during COVID-19 and reentry were involuntary due to safety and security concerns (Munthali et al., 2021; Zhang et al., 2022). Therefore, although forced and involuntary migration have been proven to have direct and indirect impacts on migrants depression (Hwang et al., 2007; Hwang W. C. et al., 2010), they already knew clearly about Chinese society’s sociopolitical landscape shifts during their absence (more nationalism, traditionalism, and centralism), and the intended environment they want to live in (foreign). Consequently, their expectation for their reentry could be lower than other identity clusters; Also, while living in China is not fitting for them, they may already working on their future plans (such as emigrating) more concretely compared to the navigators, or by the time they took the survey, some of them might have already joined the wave of new “brain drain” noticed in various reports and academic discussions (Bloomberg News, 2023; Wong and Yan, 2023; WSJ, 2023), emigrating to foreign countries (as explained earlier, the inclusion criteria includes those who left China after their reentry). Thus, with clearer inner values and preferences, wayfarers are less likely to be under the pressure of having to balance out conflicting values compared to the case of navigators.

### 4.4 Implications

The findings underscore the critical need for tailored mental health support and interventions for returnees, highlighting the role of identity clusters and socio-cultural factors associated with depression. Policymakers and mental health professionals should consider these insights in developing targeted strategies to support the mental well-being of returnees, emphasizing the importance of understanding individual and cultural dynamics in addressing mental health challenges in the context of global mobility and crises. Given the sensitive nature of transnational identity clusters, it is advised that online intervention programs that are group/identity cluster-based can be implemented to promote mental health conditions among vulnerable populations. Prior programs such as the Be Well Plan Program have proven the effectiveness of such an intervention (Fassnacht et al., 2022).

Since education attainment has been proven to be moderately negatively associated with depression, for navigators and wayfarers, education programs for returnees and even their parents should be offered by the local community and workplace. Developing bicultural integration workshops and programs focusing on leveraging their dual cultural competencies in both personal and professional contexts might lower their depression and smoothen their reacculturation; Also, considering the current sociopolitical landscape both domestic and abroad, policies and regulations that recognize and support the unique contributions of bicultural individuals, including anti-discrimination policies and initiatives that promote cultural diversity and inclusion in the workplace and societies should be promoted and implemented to mitigate the pain caused by antagonization, polarization, and intolerance. The benefits of cultural integration programs and policies in facilitating returnees’ adjustment and maximizing their contributions to society are well-documented (Ahmed, 2023).

Moreover, Chinese policies promoting international collaboration might consider granting dual citizenship to Chinese persons who are well-adjusted to both domestic and overseas environments. Bicultural individuals who have extensive experience living in a foreign country play a crucial role in enhancing mutual understanding between Eastern and Western cultures. They are also associated with enhanced creativity, problem-solving skills, and professional success in multicultural environments, which are essential for China to achieve national prosperity.

### 4.5 Limitations

The study acknowledges several limitations. First, the dynamic nature of returnees’ identity clusters suggests a need for qualitative research to uncover detailed insights into identity formation, shifts, and their correlation with depression. In response, the authors are conducting ongoing qualitative studies aiming to illuminate the complexities of returnee psychology. Furthermore, given current China’s sociopolitical context, and the consideration that a few participants in the study have multiple residencies in different countries, additional research within the diaspora could provide a richer understanding of the experiences of navigators and wayfarers in their post-emigration life. Comparative studies across cultures with similar sociocultural values, such as those in the East Asia Sphere, are also recommended to contextualize returnee depression more broadly. Also, prior research indicated that other factors about one’s mental states, such as parental COVID-19 anxiety, personal fear of COVID-19, and religious coping, are associated with depression (Yıldırım et al., 2022; Yıldırım and Çiçek, 2022), these factors are potential targets in follow-up studies among Chinese returnees. Therefore, more longitudinal studies to track the long-term impact of acculturation on mental health or intervention studies are needed to evaluate the effectiveness of targeted support programs.

## Data availability statement

The data used in the study can be found at https://osf.io/vz425/.

## Ethics statement

The studies involving human participants were reviewed and approved by the Institutional Review Board at China University of Political Science and Law. The studies were conducted in accordance with the local legislation and institutional requirements. The participants provided their written informed consent to participate in this study.

## Author contributions

RJ: Writing – original draft, Conceptualization. JW: Writing – original draft, Validation. XW: Writing – original draft, Data curation. T-TL: Writing – review & editing, Writing – original draft, Conceptualization.
